# Supplementary material for: A single WNT enhancer drives specification and regeneration of the Drosophila wing
Source: Nat Commun. 2022 Aug 22;13:4794. doi: 10.1038/s41467-022-32400-2 (PMC9395397; doi:10.1038/s41467-022-32400-2)
Supplement: Supplementary file 1 — Supplementary Information [file 41467_2022_32400_MOESM1_ESM.pdf]

Supplementary Figures

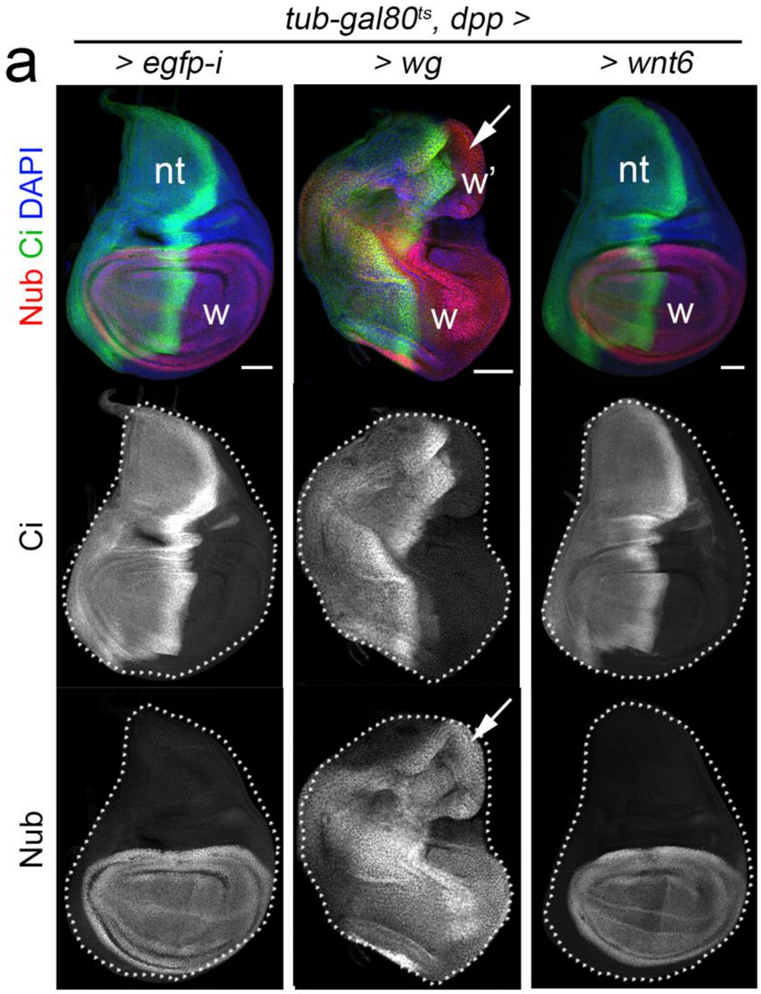

Supplementary Figure 1. Ectopic Wg, and not Wnt6, induces wing fate specification.

(**a**) Third instar wing discs expressing the indicated transgenes under the control of the *dpp-gal4* driver and stained for Nubbin (Nub, red or white), Ci (green or white) and DAPI (blue). The presumptive wing (w) and notum (nt) region are labelled and the induction of an ectopic wing (w') by Wg expression is marked by an arrow. Scale bars, 50 µm.

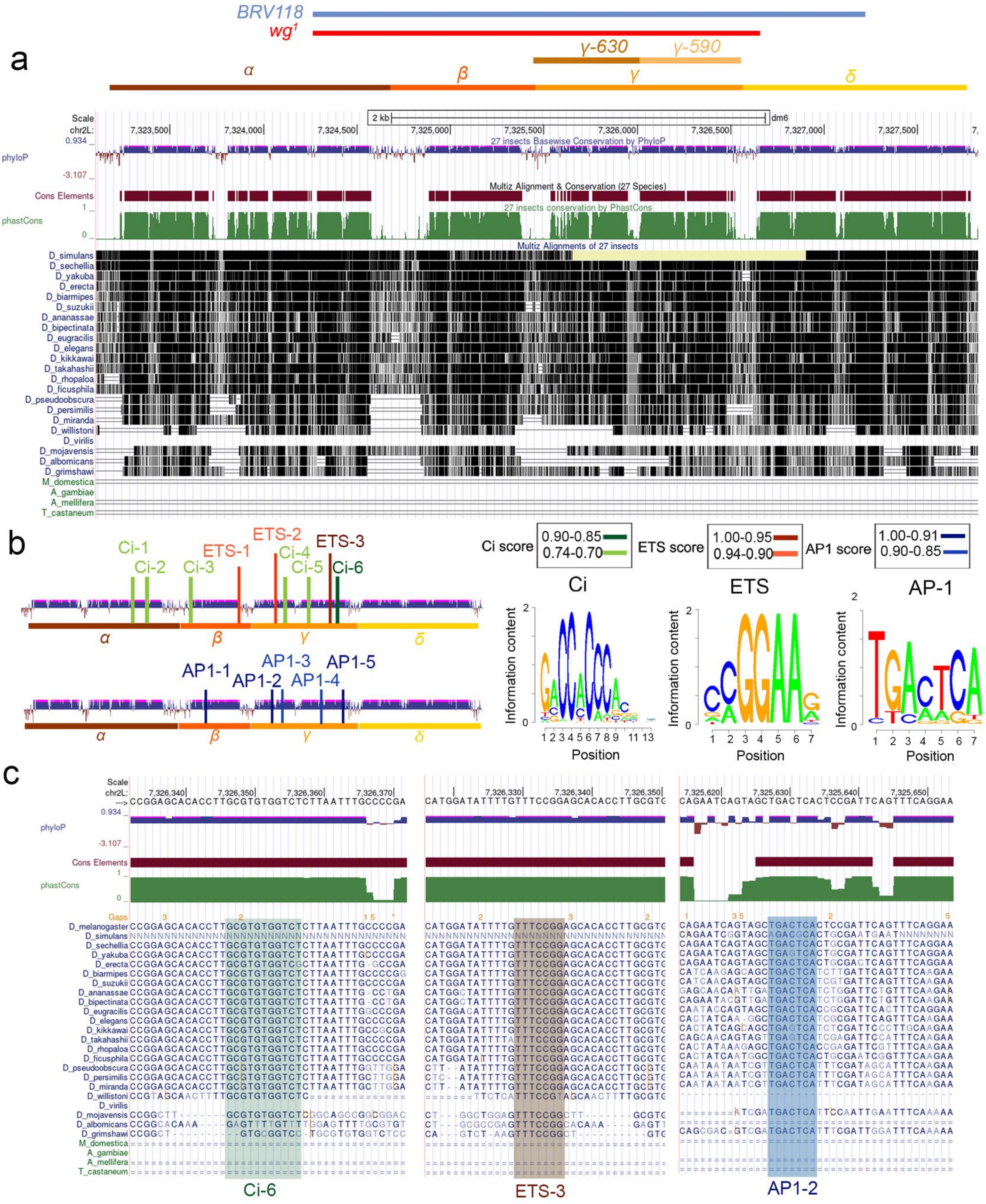
a
BRV118
wg1
γ-630
γ-590
α
β
γ
δ
Scale
chr2L:
2 kb
dm6
7,323,500
7,324,000
7,324,500
7,325,000
7,325,500
7,326,000
7,326,500
7,327,000
7,327,500
27 insects Basewise Conservation by PhyloP
phyloP
0.934
-3.107
Multiz Alignment & Conservation (27 Species)
Cons Elements
27 insects conservation by PhastCons
phastCons
Multiz Alignments of 27 insects
D_simulans
D_sechellia
D_yakuba
D_erecta
D_biarmipes
D_suzukii
D_ananassae
D_bipectinata
D_eugracilis
D_elegans
D_kikkawai
D_takahashii
D_rhopaloa
D_ficusphila
D_pseudoobscura
D_persimilis
D_miranda
D_willistoni
D_virilis
D_mojavensis
D_albomicans
D_grimshawi
M_domestica
A_gambiae
A_mellifera
T_castaneum
b
Ci-1
Ci-2
Ci-3
ETS-1
ETS-2
Ci-4
Ci-5
ETS-3
Ci-6
AP1-1
AP1-2
AP1-3
AP1-4
AP1-5
Ci score
0.90-0.85
0.74-0.70
ETS score
1.00-0.95
0.94-0.90
AP1 score
1.00-0.91
0.90-0.85
Ci
ETS
AP-1
Information content
Position
c
Scale
chr2L:
7,326,340
7,326,350
7,326,360
7,326,370
7,326,330
7,325,620
7,325,630
7,325,640
7,325,650
D_melanogaster
Gaps
Ci-6
ETS-3
AP1-2

Supplementary Figure 2. Conservation of CRMs and transcription factor binding sites.

(**a**) Cartoon depicting the CRMs spanning the *wg*[1]-enhancer and their conservation as shown by multiple alignments of 27 insect species (**b**) Cartoon depicting the CRMs spanning the *wg*[1]-enhancer and the presence of bioinformatically predicted Ci, ETS and AP1 binding sites (left) with the scores shown in the tables (top right) according to the position weighted matrices shown in the graphs (bottom right) and the evolutionary conservation shown in **c**. (**c**) Conservation of Ci, ETS and AP1 binding sites as shown by multiple alignments of 27 insect species. In **a** and **c**, tracks (purple and green) show measurements of evolutionary conservation using two methods (phastCons and phyloP) from the PHAST package (http://compgen.cshl.edu/phast/), for all 27 species. Conserved elements (brown) identified by phastCons are also displayed. Data are taken from the UCSC genome browser (https://genome-euro.ucsc.edu/index.html).

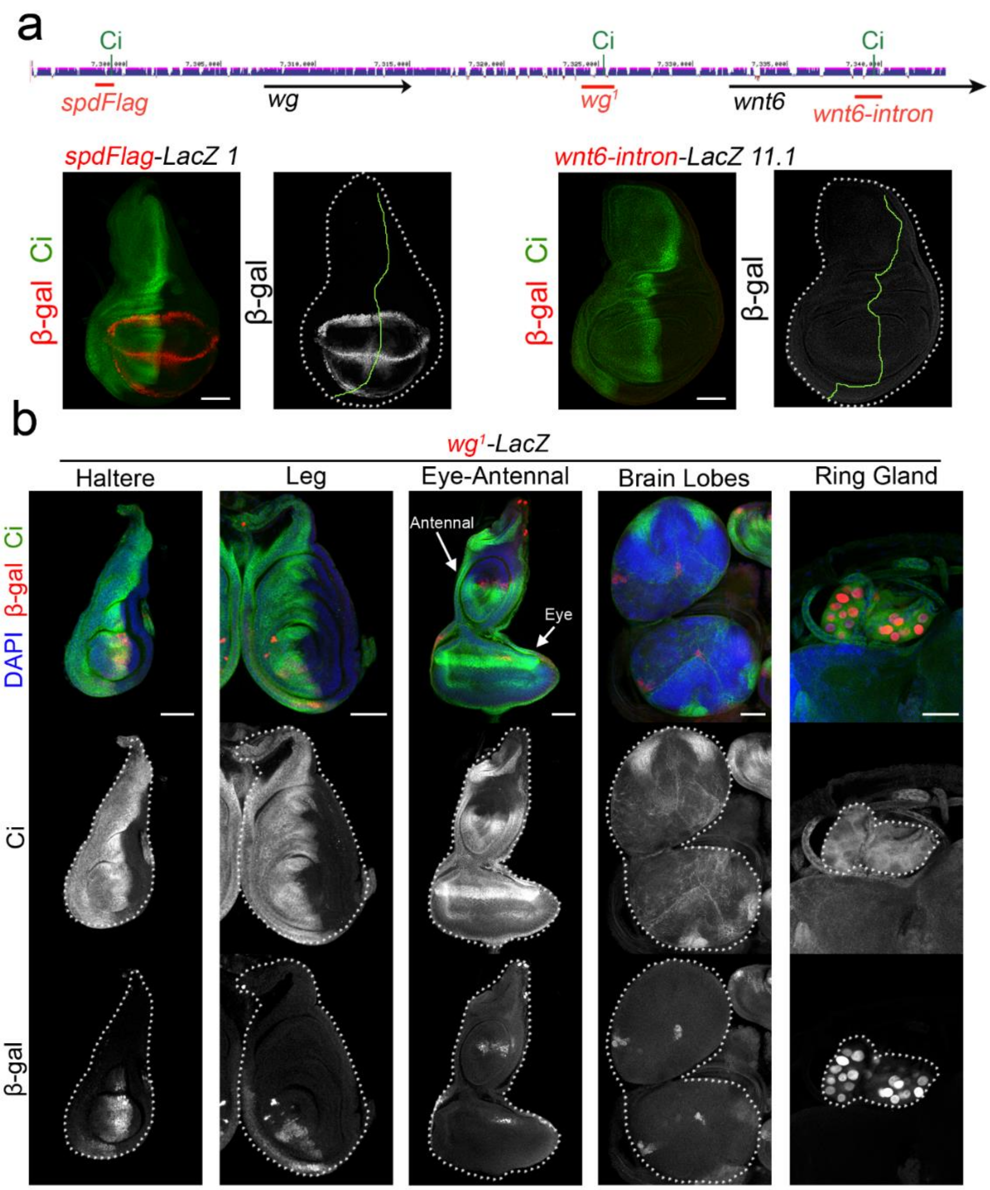

Supplementary Figure 3. The *wg*^{1}-enhancer is active in other tissues.

(**a**) Cartoon depicting the genomic location of the *spdflag*, *wg*^{1} and *wnt6*-intron enhancers with respect to the *wg* and the *wnt6* genes, and third instar wing discs bearing the *spdFlag-lacZ* and *wnt6-intron-lacZ* reporter and stained for ß-galactosidase (red or white) and Ci (green). The anterior-posterior boundary is marked by a green line. (**b**) Third instar haltere, leg and eye-antenna discs, brain lobes and ring gland of larvae bearing the *wg*^{1}*-lacZ* reporter and stained for ß-galactosidase (red or white), Ci (green or white), and DAPI (blue). Scale bars, 50 µm.

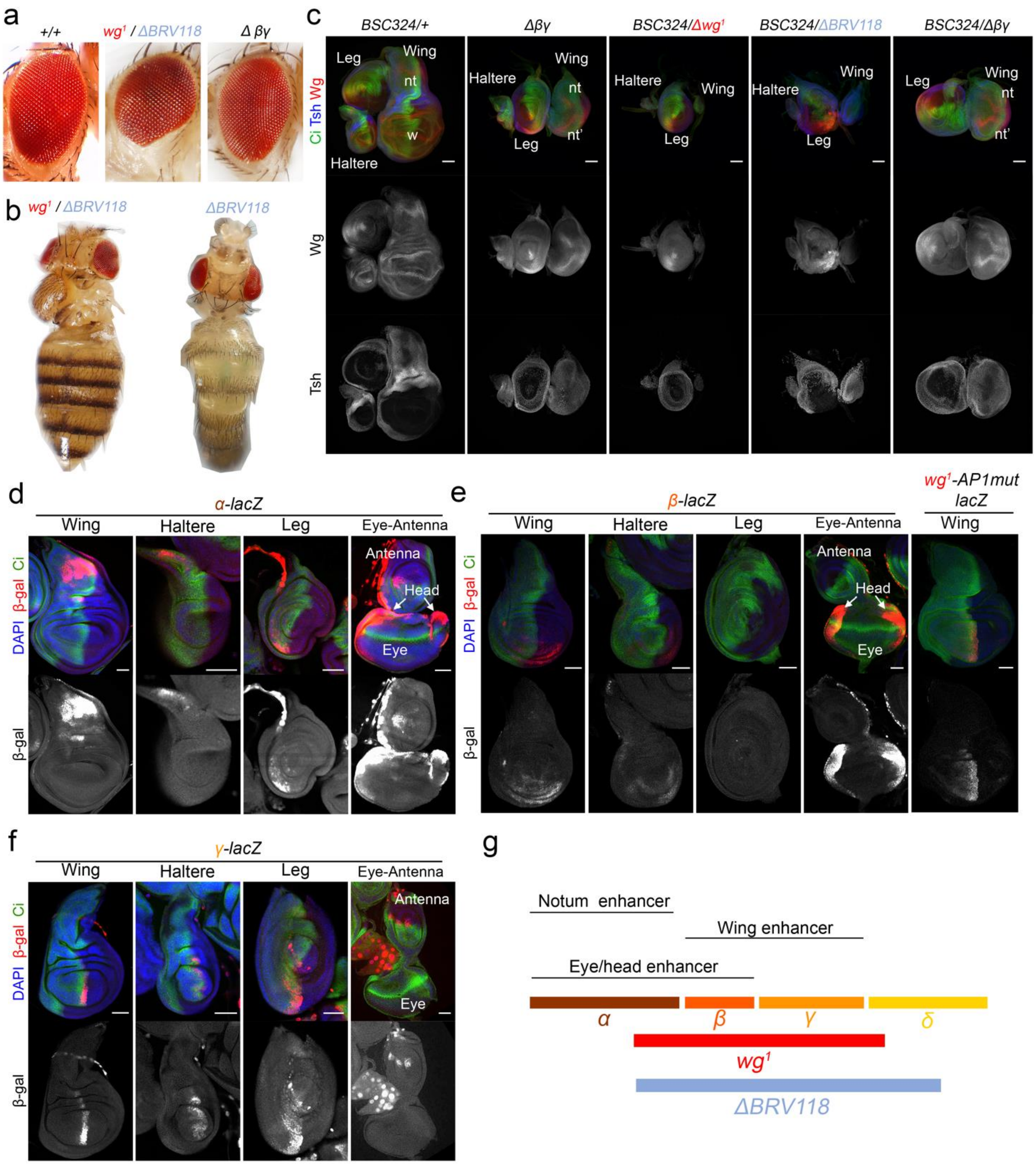

Supplementary Figure 4. Three distinct overlapping enhancers are required for the development of notum, eyes and wings.

(**a**) Adult eyes of individuals of the indicated genotypes. Note a reduction in eye size in *ΔBRV118/Δwg*^{1} but not in *Δ*βγ homozygous flies. (**b**) Examples of loss of notum structures in *ΔBRV118/Δwg*^{1} or *ΔBRV118* homozygous flies. This phenotype was not observed in *Δ*βγ homozygous flies. (**c**-**f**) Late third instar imaginal discs of the indicated genetoypes (**c**) or bearing the indicated lacZ reporters (**d**-**e**) and stained for Wg (red or white, **c**), ß-galactosidase (red or white, **d**-**f**), Teashirt (Tsh, blue or white, **c**), Ci (green), and DAPI (blue). In **c**, the wing (w) and notum (nt) presumptive regions in wing discs are labelled. Note the drastic reduction in wing and haltere disc size of some *Df(BSC324)/Δwg*^{1} and *Df(BSC324)/ΔBRV118* larvae. In contrast, *Df(BSC324)/Δ*βγ or *Δ*βγ homozygous flies showed only the presence of a duplicated notum (nt'). Note in **d**-**f**, lacZ expression driven by *Alpha* and *Beta*, but not *Gamma*, in the presumptive region of the head within the eye-antenna imaginal disc. Only *Alpha* drives expression of lacZ in the notum region. The anterior-posterior boundary is marked in **d**-**f** by a green line. (**g**) Cartoon showing the localisation of the notum, eye/head and wing enhancers according to the expression patterns driven by *Alpha*, *Beta* and *Gamma* and the imaginal and adult phenotypes. Scale bars, 50 µm.

Supplementary Tables

wg1 enhancer genomic positions

	FlyBase 1.6 Chrm 2L:		
wg Region	Start Number:	End Number:	Base Pairs:
wg gene	7.307.159	7.316.265	9.106
wnt6	7.333.714	7.352.542	18.828
wg^{1}	7.324.252	7.326.668	2.416
BRV118	7.324.275	7.327.235	2.960
BRV-A	7.324.275	7.325.250	975
BRV-B	7.325.250	7.326.235	985
BRV-C	7.326.172	7.327.303	1.131
wg-α	7.323.135	7.324.749	1.614
wg-β	7.324.770	7.325.443	673
wg-γ	7.325.445	7.326.565	1.120
wg-γ 590	7.325.993	7.326.565	572
wg-γ 630	7.325.445	7.325.993	548
wg- δ	7.326.565	7.327.855	1.290
Df(2L)BSC226 (Center)	7.249.632	7.366.119	116.487
Df(2L)BSC291 (Left)	7.042.642	7.366.119	323.477
Df(2L)BSC324 (Right)	7.249.633	7.718.010	468.377
BRV-AC59 Hariharan Deletion ABC	7.324.259	7.327.303	3.044
Df(2L)Exel8019	7.140.259	7.202.317	62.058
Spd^{flg}	7.297.496	7.298.924	1.428
Spd^{flg} Ci Binding	7.298.788	7.298.799	11
wnt6 intron	7.338.026	7.339.980	1.954
wnt6 Ci Binding	7.339.563	7.339.574	11

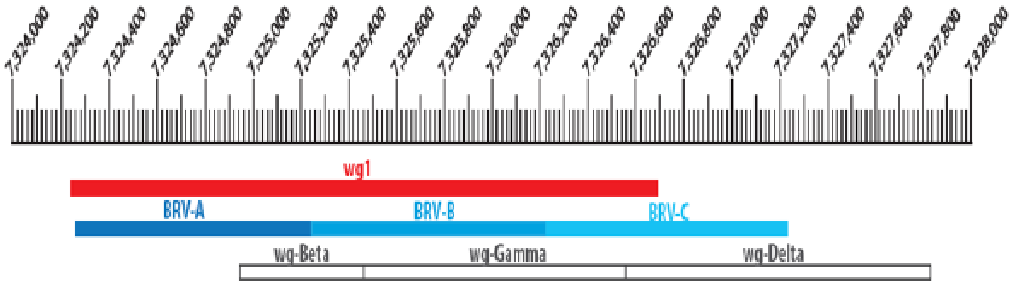

Supplementary Table 1. Genomic coordinates.

Table containing information on the genomic position of the regions analyzed in this work around, overlapping or within the *wg^{1}*-enhancer.

Ci-predicted binding sites

seqnames	start	end	width	strand	score.avg	score.max	cons.avg	cons.max	pv.min	pv.max	TF	merged BS	region	nr-Fig. S2b
chr2L	7324379	7324390	12	*	0,71	**0,71**	1	1	0,0016605	0,00166	MEME_fly_factor_survey-ci_FBgn0004859	Ci BS α1	α	Ci-1*
chr2L	7324486	7324499	14	*	0,74	**0,74**	0,999143	0,999143	0,00086389	0,00086	MEME_fly_factor_survey-ci_FBgn0004859_2	Ci BS α2		Ci-2*
chr2L	7324904	7324915	12	*	0,73	**0,73**	0,999833	0,999833	0,00108345	0,00108	MEME_fly_factor_survey-ci_FBgn0004859	Ci BS β1	β	Ci-3*
chr2L	7325793	7325815	23	*	0,735	**0,74**	0,958214	0,998786	0,00086389	0,00107	MEME_fly_factor_survey-ci_FBgn0004859_2	Ci BS γ1	γ	Ci-4*
chr2L	7325796	7325812	17	*	0,72	**0,74**	0,973125	0,9985	0,0008713	0,00204	MEME_fly_factor_survey-ci_FBgn0004859			
chr2L	7326182	7326195	14	*	0,7	**0,7**	0,994429	0,994429	0,00199642	0,002	MEME_fly_factor_survey-ci_FBgn0004859_2	Ci BS γ2		Ci-5*
chr2L	7326185	7326196	12	*	0,74	**0,74**	0,980667	0,980667	0,0008713	0,00087	MEME_fly_factor_survey-ci_FBgn0004859			
chr2L	7326343	7326356	14	*	0,82	**0,82**	1	1	0,00012201	0,00012	MEME_fly_factor_survey-ci_FBgn0004859_2	Ci BS γ3		
chr2L	7326346	7326359	14	*	0,785	**0,86**	0,99825	1	3,6678E-05	0,00166	MEME_fly_factor_survey-ci_FBgn0004859			Ci-6* (Ci-mut)

*Depicted in Fig 2, 3, 4

Region	Start	End	Num bp
α	7323135	7324749	1614
β	7324770	7325443	673
γ	7325445	7326565	1120
δ	7326565	7327855	1290

Ci Score
0,90-0,85
0,84-0,80
0,79-0,75
0,74-0,70

Supplementary Table 2. Ci-predicted binding sites.

Table containing information on the bioinformatically-predicted Ci binding sites identified in this enhancer (genomic coordinates and statistical parameters on conservation and match).

ETS-predicted binding sites

seqnames	start	end	width	strand	score.avg	score.max	cons.avg	cons.max	pv.min	pv.max	TF	Merged BS	region	nr-Fig S2b
chr2L	7324347	7324356	10	*	0,75	**0,75**	1	1	0,002029	0,002029	MEME_fly_factor_survey-pointed_FBgn0003118	ETS BS α1	α	
chr2L	7324397	7324406	10	*	0,74	**0,74**	0,9982	0,9982	0,002373	0,002373	MEME_fly_factor_survey-pointed_FBgn0003118	ETS BS α2		
chr2L	7324411	7324418	8	*	0,74	**0,74**	0,934	0,934	0,005392	0,005392	Jaspar_2020_MA0026.1-Eip74EF	ETS BS α3		
chr2L	7324468	7324477	10	*	0,7	**0,7**	1	1	0,003748	0,003748	MEME_fly_factor_survey-pointed_FBgn0003118	ETS BS α4		
chr2L	7324514	7324525	12	*	0,735	**0,76**	1	1	0,004224	0,01005	Jaspar_2020_MA0026.1-Eip74EF	ETS BS α5		
chr2L	7324555	7324564	10	*	0,87	**0,87**	1	1	0,000117	0,000117	MEME_fly_factor_survey-yan_FBgn0000097	ETS BS α6		
chr2L	7324555	7324564	10	*	0,88	**0,88**	1	1	0,000127	0,000127	MEME_fly_factor_survey-pointed_FBgn0003118			
chr2L	7324556	7324563	8	*	0,83	**0,83**	1	1	0,001654	0,001654	Jaspar_2020_MA0026.1-Eip74EF			
chr2L	7324568	7324575	8	*	0,74	**0,74**	0,70975	0,70975	0,005392	0,005392	Jaspar_2020_MA0026.1-Eip74EF	ETS BS α7		
chr2L	7325021	7325033	13	*	0,78	**0,83**	0,999438	0,99975	0,001654	0,007114	Jaspar_2020_MA0026.1-Eip74EF	ETS BS β1	β	
chr2L	7325057	7325066	10	*	0,71	**0,71**	0,9966	0,9966	0,003688	0,003688	MEME_fly_factor_survey-yan_FBgn0000097	ETS BS β1		
chr2L	7325057	7325066	10	*	0,74	**0,74**	0,9966	0,9966	0,002373	0,002373	MEME_fly_factor_survey-pointed_FBgn0003118			
chr2L	7325093	7325102	10	*	0,77	**0,77**	1	1	0,001136	0,001136	MEME_fly_factor_survey-yan_FBgn0000097	ETS BS β2		
chr2L	7325093	7325102	10	*	0,79	**0,79**	1	1	0,000819	0,000819	MEME_fly_factor_survey-pointed_FBgn0003118			
chr2L	7325094	7325101	8	*	0,78	**0,78**	1	1	0,003371	0,003371	Jaspar_2020_MA0026.1-Eip74EF			
chr2L	7325147	7325156	10	*	0,71	**0,71**	0,9475	0,9475	0,003278	0,003278	MEME_fly_factor_survey-pointed_FBgn0003118	ETS BS β3		
chr2L	7325148	7325155	8	*	0,78	**0,78**	0,9775	0,9775	0,003371	0,003371	Jaspar_2020_MA0026.1-Eip74EF			
chr2L	7325171	7325180	10	*	0,74	**0,74**	0,9106	0,9106	0,002373	0,002373	MEME_fly_factor_survey-pointed_FBgn0003118	ETS BS β4		
chr2L	7325171	7325180	10	*	0,84	**0,84**	0,9106	0,9106	0,000234	0,000234	MEME_fly_factor_survey-yan_FBgn0000097			
chr2L	7325172	7325179	8	*	0,91	**0,91**	0,920125	0,920125	0,000227	0,000227	Jaspar_2020_MA0026.1-Eip74EF			**ETS-1***
chr2L	7325204	7325211	8	*	0,73	**0,73**	1	1	0,007114	0,007114	Jaspar_2020_MA0026.1-Eip74EF	ETS BS β5		
chr2L	7325280	7325289	10	*	0,78	**0,78**	1	1	0,001012	0,001012	MEME_fly_factor_survey-pointed_FBgn0003118	ETS BS β6		
chr2L	7325280	7325289	10	*	0,8	**0,8**	1	1	0,000637	0,000637	MEME_fly_factor_survey-yan_FBgn0000097			
chr2L	7325281	7325288	8	*	0,83	**0,83**	1	1	0,001654	0,001654	Jaspar_2020_MA0026.1-Eip74EF			
chr2L	7325361	7325368	8	*	0,82	**0,82**	0,999	0,999	0,001682	0,001682	Jaspar_2020_MA0026.1-Eip74EF	ETS BS β7		
chr2L	7325644	7325656	13	*	0,705	**0,71**	0,9216	1	0,003688	0,004226	MEME_fly_factor_survey-yan_FBgn0000097	ETS BS γ1	γ	
chr2L	7325645	7325655	11	*	0,81	**0,84**	0,951062	1	0,000896	0,003371	Jaspar_2020_MA0026.1-Eip74EF			
chr2L	7325647	7325656	10	*	0,74	**0,74**	1	1	0,002373	0,002373	MEME_fly_factor_survey-pointed_FBgn0003118			
chr2L	7325656	7325663	8	*	0,71	**0,71**	1	1	0,01005	0,01005	Jaspar_2020_MA0026.1-Eip74EF	ETS BS γ2		
chr2L	7325720	7325733	14	*	0,775	**0,82**	0,9981	1	0,000375	0,002562	MEME_fly_factor_survey-yan_FBgn0000097	ETS BS γ3		
chr2L	7325721	7325738	18	*	0,813333	**0,9**	0,979125	1	0,000317	0,01005	Jaspar_2020_MA0026.1-Eip74EF			**ETS-2***
chr2L	7325724	7325733	10	*	0,76	**0,76**	0,9962	0,9962	0,001823	0,001823	MEME_fly_factor_survey-pointed_FBgn0003118			
chr2L	7325825	7325834	10	*	0,81	**0,81**	0,9982	0,9982	0,000465	0,000465	MEME_fly_factor_survey-yan_FBgn0000097	ETS BS γ4		
chr2L	7325825	7325834	10	*	0,87	**0,87**	0,9982	0,9982	0,000194	0,000194	MEME_fly_factor_survey-pointed_FBgn0003118			
chr2L	7325826	7325836	11	*	0,745	**0,78**	0,999125	1	0,003371	0,01005	Jaspar_2020_MA0026.1-Eip74EF			
chr2L	7325843	7325850	8	*	0,71	**0,71**	0,99625	0,99625	0,01005	0,01005	Jaspar_2020_MA0026.1-Eip74EF	ETS BS γ5		
chr2L	7325856	7325863	8	*	0,77	**0,77**	1	1	0,003551	0,003551	Jaspar_2020_MA0026.1-Eip74EF	ETS BS γ6		
chr2L	7325879	7325888	10	*	0,74	**0,74**	1	1	0,002024	0,002024	MEME_fly_factor_survey-yan_FBgn0000097	ETS BS γ7		
chr2L	7325880	7325887	8	*	0,78	**0,78**	1	1	0,003371	0,003371	Jaspar_2020_MA0026.1-Eip74EF			
chr2L	7326069	7326078	10	*	0,73	**0,73**	1	1	0,002562	0,002562	MEME_fly_factor_survey-yan_FBgn0000097	ETS BS γ8		
chr2L	7326069	7326078	10	*	0,77	**0,77**	1	1	0,001505	0,001505	MEME_fly_factor_survey-pointed_FBgn0003118			
chr2L	7326070	7326077	8	*	0,78	**0,78**	1	1	0,003371	0,003371	Jaspar_2020_MA0026.1-Eip74EF			
chr2L	7326078	7326090	13	*	0,735	**0,74**	1	1	0,005392	0,007114	Jaspar_2020_MA0026.1-Eip74EF	ETS BS γ9		
chr2L	7326082	7326091	10	*	0,71	**0,71**	1	1	0,003688	0,003688	MEME_fly_factor_survey-yan_FBgn0000097	ETS BS γ10		
chr2L	7326123	7326130	8	*	0,73	**0,73**	0,9995	0,9995	0,007114	0,007114	Jaspar_2020_MA0026.1-Eip74EF	ETS BS γ11		
chr2L	7326165	7326172	8	*	0,71	**0,71**	0,984875	0,984875	0,01005	0,01005	Jaspar_2020_MA0026.1-Eip74EF	ETS BS γ12		
chr2L	7326173	7326180	8	*	0,76	**0,76**	0,99925	0,99925	0,004224	0,004224	Jaspar_2020_MA0026.1-Eip74EF	ETS BS γ13		
chr2L	7326328	7326337	10	*	0,82	**0,82**	1	1	0,000307	0,000307	MEME_fly_factor_survey-pointed_FBgn0003118	ETS BS γ14		
chr2L	7326328	7326337	10	*	0,83	**0,83**	1	1	0,000288	0,000288	MEME_fly_factor_survey-yan_FBgn0000097			
chr2L	7326329	7326339	11	*	0,845	**0,95**	1	1	9,66E-05	0,005392	Jaspar_2020_MA0026.1-Eip74EF			**ETS-3***
chr2L	7326343	7326350	8	*	0,73	**0,73**	1	1	0,007114	0,007114	Jaspar_2020_MA0026.1-Eip74EF	ETS BS γ15		
chr2L	7326388	7326398	11	*	0,715	**0,73**	0,87995	0,9012	0,002562	0,004226	MEME_fly_factor_survey-yan_FBgn0000097	ETS BS γ16		
chr2L	7326389	7326398	10	*	0,74	**0,74**	0,8587	0,8587	0,002373	0,002373	MEME_fly_factor_survey-pointed_FBgn0003118			
chr2L	7326389	7326397	9	*	0,76	**0,78**	0,933937	0,991375	0,003371	0,005392	Jaspar_2020_MA0026.1-Eip74EF			
chr2L	7326432	7326441	10	*	0,72	**0,72**	1	1	0,002776	0,002776	MEME_fly_factor_survey-yan_FBgn0000097	ETS BS γ17		
chr2L	7326433	7326440	8	*	0,73	**0,73**	1	1	0,007114	0,007114	Jaspar_2020_MA0026.1-Eip74EF			

*Depicted in Fig 2, 3,

Region	Start	End	Num bp
α	7323135	7324749	1614
β	7324770	7325443	673
γ	7325445	7326565	1120
δ	7326565	7327855	1290

ETS Score
1-0,95
0,94-0,90
0,89-0,85
0,84-0,80
0,79-0,75
0,74-0,70

Supplementary Table 3. ETS-predicted binding sites.

Table containing information on the bioinformatically-predicted ETS binding sites identified in this enhancer (genomic coordinates and statistical parameters on conservation and match).

Ci & ETS-predicted binding sites

end	width	strand	score.avg	score.max	cons.avg	cons.max	pv.min	pv.max	TF	Merged BS	region
7324356	10	*	0,75	**0,75**	1	1	0,00202891	0,002028914	MEME_fly_factor_survey-pointed_FBgn0003118	ETS BS α1	α
7324390	12	*	0,71	**0,71**	1	1	0,0016605	0,001660498	MEME_fly_factor_survey-ci_FBgn0004859	Ci BS α1	
7324406	10	*	0,74	**0,74**	0,998199999	0,9982	0,0023733	0,0023733	MEME_fly_factor_survey-pointed_FBgn0003118	ETS BS α2	
7324418	8	*	0,74	**0,74**	0,934	0,934	0,00539244	0,005392442	Jaspar_2020_MA0026.1-Eip74EF	ETS BS α3	
7324477	10	*	0,7	**0,7**	1	1	0,00374811	0,003748112	MEME_fly_factor_survey-pointed_FBgn0003118	ETS BS α4	
7324499	14	*	0,74	**0,74**	0,999142864	0,99914286	0,00086389	0,000863893	MEME_fly_factor_survey-ci_FBgn0004859_2	Ci BS α2	
7324525	12	*	0,735	**0,76**	1	1	0,00422431	0,010049866	Jaspar_2020_MA0026.1-Eip74EF	ETS BS α5	
7324564	10	*	0,87	**0,87**	1	1	0,00011746	0,000117457	MEME_fly_factor_survey-yan_FBgn0000097	ETS BS α6	
7324564	10	*	0,88	**0,88**	1	1	0,00012691	0,000126912	MEME_fly_factor_survey-pointed_FBgn0003118		
7324563	8	*	0,83	**0,83**	1	1	0,00165395	0,001653948	Jaspar_2020_MA0026.1-Eip74EF		
7324575	8	*	0,74	**0,74**	0,709749997	0,70975	0,00539244	0,005392442	Jaspar_2020_MA0026.1-Eip74EF	ETS BS α7	
7324915	12	*	0,73	**0,73**	0,999833335	0,99983334	0,00108345	0,00108345	MEME_fly_factor_survey-ci_FBgn0004859	Ci BS β1	β
7325033	13	*	0,78	**0,83**	0,999437504	0,99975	0,00165395	0,007113993	Jaspar_2020_MA0026.1-Eip74EF	ETS BS β1	
7325066	10	*	0,71	**0,71**	0,996600002	0,9966	0,00368844	0,003688438	MEME_fly_factor_survey-yan_FBgn0000097	ETS BS β1	
7325066	10	*	0,74	**0,74**	0,996600002	0,9966	0,0023733	0,0023733	MEME_fly_factor_survey-pointed_FBgn0003118		
7325102	10	*	0,77	**0,77**	1	1	0,0011357	0,001135696	MEME_fly_factor_survey-yan_FBgn0000097	ETS BS β2	
7325102	10	*	0,79	**0,79**	1	1	0,00081947	0,000819466	MEME_fly_factor_survey-pointed_FBgn0003118		
7325101	8	*	0,78	**0,78**	1	1	0,00337067	0,00337067	Jaspar_2020_MA0026.1-Eip74EF		
7325156	10	*	0,71	**0,71**	0,947499996	0,9475	0,00327765	0,003277654	MEME_fly_factor_survey-pointed_FBgn0003118	ETS BS β3	
7325155	8	*	0,78	**0,78**	0,977499999	0,9775	0,00337067	0,00337067	Jaspar_2020_MA0026.1-Eip74EF		
7325180	10	*	0,74	**0,74**	0,910600001	0,9106	0,0023733	0,0023733	MEME_fly_factor_survey-pointed_FBgn0003118	ETS BS β4	
7325180	10	*	0,84	**0,84**	0,910600001	0,9106	0,00023386	0,000233863	MEME_fly_factor_survey-yan_FBgn0000097		
7325179	8	*	0,91	**0,91**	0,920125	0,920125	0,00022737	0,000227371	Jaspar_2020_MA0026.1-Eip74EF		
7325211	8	*	0,73	**0,73**	1	1	0,00711399	0,007113993	Jaspar_2020_MA0026.1-Eip74EF	ETS BS β5	
7325289	10	*	0,78	**0,78**	1	1	0,00101152	0,001011515	MEME_fly_factor_survey-pointed_FBgn0003118	ETS BS β6	
7325289	10	*	0,8	**0,8**	1	1	0,00063708	0,000637082	MEME_fly_factor_survey-yan_FBgn0000097		
7325288	8	*	0,83	**0,83**	1	1	0,00165395	0,001653948	Jaspar_2020_MA0026.1-Eip74EF		
7325368	8	*	0,82	**0,82**	0,999000013	0,99900001	0,00168166	0,001681661	Jaspar_2020_MA0026.1-Eip74EF	ETS BS β7	
7325656	13	*	0,705	**0,71**	0,9216	1	0,00368844	0,004225714	MEME_fly_factor_survey-yan_FBgn0000097	ETS BS γ1	γ
7325655	11	*	0,81	**0,84**	0,9510625	1	0,00089647	0,00337067	Jaspar_2020_MA0026.1-Eip74EF		
7325656	10	*	0,74	**0,74**	1	1	0,0023733	0,0023733	MEME_fly_factor_survey-pointed_FBgn0003118		
7325663	8	*	0,71	**0,71**	1	1	0,01004987	0,010049866	Jaspar_2020_MA0026.1-Eip74EF	ETS BS γ2	
7325733	14	*	0,775	**0,82**	0,998100001	1	0,00037506	0,002562197	MEME_fly_factor_survey-yan_FBgn0000097	ETS BS γ3	
7325738	18	*	0,81333333	**0,9**	0,979125003	1	0,00031723	0,010049866	Jaspar_2020_MA0026.1-Eip74EF		
7325733	10	*	0,76	**0,76**	0,996200001	0,9962	0,00182321	0,001823207	MEME_fly_factor_survey-pointed_FBgn0003118		
7325815	23	*	0,735	**0,74**	0,958214289	0,99878572	0,00086389	0,001069334	MEME_fly_factor_survey-ci_FBgn0004859_2	Ci BS γ1	
7325812	17	*	0,72	**0,74**	0,973125006	0,9985	0,0008713	0,002036861	MEME_fly_factor_survey-ci_FBgn0004859		
7325834	10	*	0,81	**0,81**	0,998199999	0,9982	0,00046499	0,000464995	MEME_fly_factor_survey-yan_FBgn0000097	ETS BS γ4	
7325834	10	*	0,87	**0,87**	0,998199999	0,9982	0,00019415	0,00019415	MEME_fly_factor_survey-pointed_FBgn0003118		
7325836	11	*	0,745	**0,78**	0,999125	1	0,00337067	0,010049866	Jaspar_2020_MA0026.1-Eip74EF		
7325850	8	*	0,71	**0,71**	0,996249996	0,99625	0,01004987	0,010049866	Jaspar_2020_MA0026.1-Eip74EF	ETS BS γ5	
7325863	8	*	0,77	**0,77**	1	1	0,0035508	0,003550803	Jaspar_2020_MA0026.1-Eip74EF	ETS BS γ6	
7325888	10	*	0,74	**0,74**	1	1	0,00202387	0,002023871	MEME_fly_factor_survey-yan_FBgn0000097	ETS BS γ7	
7325887	8	*	0,78	**0,78**	1	1	0,00337067	0,00337067	Jaspar_2020_MA0026.1-Eip74EF		
7326078	10	*	0,73	**0,73**	1	1	0,0025622	0,002562197	MEME_fly_factor_survey-yan_FBgn0000097	ETS BS γ8	
7326078	10	*	0,77	**0,77**	1	1	0,0015053	0,001505296	MEME_fly_factor_survey-pointed_FBgn0003118		
7326077	8	*	0,78	**0,78**	1	1	0,00337067	0,00337067	Jaspar_2020_MA0026.1-Eip74EF		
7326090	13	*	0,735	**0,74**	1	1	0,00539244	0,007113993	Jaspar_2020_MA0026.1-Eip74EF	ETS BS γ9	
7326091	10	*	0,71	**0,71**	1	1	0,00368844	0,003688438	MEME_fly_factor_survey-yan_FBgn0000097	ETS BS γ10	
7326130	8	*	0,73	**0,73**	0,999499999	0,9995	0,00711399	0,007113993	Jaspar_2020_MA0026.1-Eip74EF	ETS BS γ11	
7326172	8	*	0,71	**0,71**	0,984875001	0,984875	0,01004987	0,010049866	Jaspar_2020_MA0026.1-Eip74EF	ETS BS γ12	
7326180	8	*	0,76	**0,76**	0,999249995	0,99924999	0,00422431	0,004224307	Jaspar_2020_MA0026.1-Eip74EF	ETS BS γ13	
7326195	14	*	0,7	**0,7**	0,994428571	0,99442857	0,00199642	0,001996424	MEME_fly_factor_survey-ci_FBgn0004859_2	Ci BS γ2	
7326196	12	*	0,74	**0,74**	0,980666667	0,98066667	0,0008713	0,000871303	MEME_fly_factor_survey-ci_FBgn0004859		
7326337	10	*	0,82	**0,82**	1	1	0,0003074	0,000307405	MEME_fly_factor_survey-pointed_FBgn0003118	ETS BS γ14	
7326337	10	*	0,83	**0,83**	1	1	0,00028828	0,000288284	MEME_fly_factor_survey-yan_FBgn0000097		
7326339	11	*	0,845	**0,95**	1	1	9,6575E-05	0,005392442	Jaspar_2020_MA0026.1-Eip74EF		
7326350	8	*	0,73	**0,73**	1	1	0,00711399	0,007113993	Jaspar_2020_MA0026.1-Eip74EF	ETS BS γ15	
7326356	14	*	0,82	**0,82**	1	1	0,00012201	0,000122013	MEME_fly_factor_survey-ci_FBgn0004859_2	Ci BS γ3	
7326359	14	*	0,785	**0,86**	0,99825	1	3,6678E-05	0,001660498	MEME_fly_factor_survey-ci_FBgn0004859		
7326398	11	*	0,715	**0,73**	0,879949999	0,9012	0,0025622	0,004225714	MEME_fly_factor_survey-yan_FBgn0000097	ETS BS γ16	
7326398	10	*	0,74	**0,74**	0,858699998	0,8587	0,0023733	0,0023733	MEME_fly_factor_survey-pointed_FBgn0003118		
7326397	9	*	0,76	**0,78**	0,933937499	0,991375	0,00337067	0,005392442	Jaspar_2020_MA0026.1-Eip74EF		
7326441	10	*	0,72	**0,72**	1	1	0,00277568	0,002775679	MEME_fly_factor_survey-yan_FBgn0000097	ETS BS γ17	
7326440	8	*	0,73	**0,73**	1	1	0,00711399	0,007113993	Jaspar_2020_MA0026.1-Eip74EF		

Region	Start	End	Num bp
α	7323135	7324749	1614
β	7324770	7325443	673
γ	7325445	7326565	1120
δ	7326565	7327855	1290

Ci Score	ETS Score
0,90-0,85	1-0,95
0,84-0,80	0,94-0,90
0,79-0,75	0,89-0,85
0,74-0,70	0,84-0,80
	0,79-0,75
	0,74-0,70

Supplementary Table 4. Ci- and ETS-predicted binding sites.

Table containing information on the bioinformatically-predicted Ci and ETS binding sites identified in this enhancer (genomic coordinates and statistical parameters on conservation and match).

AP1-predicted binding sites

seqnames	start	end	width	strand	score.avg	score.max	cons.avg	cons.max	pv.min	pv.max	TF	Merged BS	region	nr-Fig S2b
chr2L	7E+06	7E+06	12	*	0,70	0,7	1	1	0,004240646	0,004240646	MEME_fly_factor_survey-Ap1_FBgn0001291	AP1 BS α1	α	
chr2L	7E+06	7E+06	12	*	0,70	0,7	1	1	0,004240646	0,004240646	MEME_fly_factor_survey-Ap1_FBgn0001297			
chr2L	7E+06	7E+06	8	*	0,70	0,7	1	1	0,011828058	0,011828058	Jaspar_2020_MA0099.2-FOS::JUN			
chr2L	7E+06	7E+06	12	*	0,70	0,7	1	1	0,004240646	0,004240646	MEME_fly_factor_survey-Ap1_FBgn0001291	AP1 BS α2		
chr2L	7E+06	7E+06	12	*	0,70	0,7	1	1	0,004240646	0,004240646	MEME_fly_factor_survey-Ap1_FBgn0001297			
chr2L	7E+06	7E+06	8	*	0,75	0,75	1	1	0,004462464	0,004462464	Jaspar_2020_MA0099.2-FOS::JUN			
chr2L	7E+06	7E+06	8	*	0,70	0,7	1	1	0,011828058	0,011828058	Jaspar_2020_MA0099.2-FOS::JUN	AP1 BS α3		
chr2L	7E+06	7E+06	9	*	0,76	0,76	1	1	0,003164505	0,003164505	Jaspar_2020_MA0099.1-JUN::FOS			
chr2L	7E+06	7E+06	8	*	0,70	0,7	1	1	0,011828058	0,011828058	Jaspar_2020_MA0099.2-FOS::JUN	AP1 BS α4		
chr2L	7E+06	7E+06	11	*	0,71	0,71	1	1	0,00338315	0,00338315	Jaspar_2020_MA0099.3-FOS::JUN	AP1 BS α5		
chr2L	7E+06	7E+06	8	*	0,71	0,71	1	1	0,009428202	0,009428202	Jaspar_2020_MA0099.2-FOS::JUN			
chr2L	7E+06	7E+06	12	*	0,75	0,76	1	1	0,001199109	0,001931829	MEME_fly_factor_survey-Ap1_FBgn0001291	AP1 BS β1	β	
chr2L	7E+06	7E+06	12	*	0,75	0,76	1	1	0,001199109	0,001931829	MEME_fly_factor_survey-Ap1_FBgn0001297			
chr2L	7E+06	7E+06	11	*	0,76	0,76	1	1	0,001243398	0,001243398	Jaspar_2020_MA0099.3-FOS::JUN			
chr2L	7E+06	7E+06	8	*	0,72	0,72	1	1	0,008114574	0,008114574	Jaspar_2020_MA0099.2-FOS::JUN			
chr2L	7E+06	7E+06	12	*	0,85	0,85	1	1	0,000126027	0,000180187	Jaspar_2020_MA0099.3-FOS::JUN	AP1 BS β2		
chr2L	7E+06	7E+06	12	*	0,84	0,85	1	1	0,000128372	0,000203394	MEME_fly_factor_survey-Ap1_FBgn0001291			
chr2L	7E+06	7E+06	12	*	0,84	0,85	1	1	0,000128372	0,000203394	MEME_fly_factor_survey-Ap1_FBgn0001297			
chr2L	7E+06	7E+06	10	*	0,87	0,89	1	1	0,00021105	0,000581117	Jaspar_2020_MA0099.1-JUN::FOS			
chr2L	7E+06	7E+06	8	*	0,91	0,93	1	1	0,000111099	0,000401536	Jaspar_2020_MA0099.2-FOS::JUN			AP1-1*
chr2L	7E+06	7E+06	8	*	0,76	0,76	0,9875	0,9875	0,003440731	0,003440731	Jaspar_2020_MA0099.2-FOS::JUN	AP1 BS β3		
chr2L	7E+06	7E+06	12	*	0,70	0,7	0,903833	0,903833	0,004240646	0,004240646	MEME_fly_factor_survey-Ap1_FBgn0001291	AP1 BS β4		
chr2L	7E+06	7E+06	12	*	0,70	0,7	0,903833	0,903833	0,004240646	0,004240646	MEME_fly_factor_survey-Ap1_FBgn0001297			
chr2L	7E+06	7E+06	9	*	0,71	0,71	0,872222	0,872222	0,007809067	0,007809067	Jaspar_2020_MA0099.1-JUN::FOS			
chr2L	7E+06	7E+06	8	*	0,70	0,7	0,85625	0,85625	0,011828058	0,011828058	Jaspar_2020_MA0099.2-FOS::JUN			
chr2L	7E+06	7E+06	9	*	0,70	0,7	1	1	0,009040818	0,009040818	Jaspar_2020_MA0099.1-JUN::FOS	AP1 BS β5		
chr2L	7E+06	7E+06	8	*	0,72	0,72	1	1	0,008114574	0,008114574	Jaspar_2020_MA0099.2-FOS::JUN			
chr2L	7E+06	7E+06	8	*	0,70	0,7	0,999875	0,999875	0,011828058	0,011828058	Jaspar_2020_MA0099.2-FOS::JUN			
chr2L	7E+06	7E+06	11	*	0,72	0,72	1	1	0,002732809	0,002732809	Jaspar_2020_MA0099.3-FOS::JUN	AP1 BS β6		
chr2L	7E+06	7E+06	9	*	0,71	0,71	1	1	0,007809067	0,007809067	Jaspar_2020_MA0099.1-JUN::FOS			
chr2L	7E+06	7E+06	8	*	0,75	0,75	1	1	0,004462464	0,004462464	Jaspar_2020_MA0099.2-FOS::JUN			
chr2L	7E+06	7E+06	12	*	0,75	0,75	0,845083	0,845083	0,001472316	0,001931829	MEME_fly_factor_survey-Ap1_FBgn0001291	AP1 BS γ1	γ	
chr2L	7E+06	7E+06	12	*	0,75	0,75	0,845083	0,845083	0,001472316	0,001931829	MEME_fly_factor_survey-Ap1_FBgn0001297			
chr2L	7E+06	7E+06	12	*	0,76	0,77	0,8775	0,921091	0,000993427	0,001850203	Jaspar_2020_MA0099.3-FOS::JUN			
chr2L	7E+06	7E+06	10	*	0,78	0,8	0,951389	0,995	0,001599528	0,003164505	Jaspar_2020_MA0099.1-JUN::FOS			
chr2L	7E+06	7E+06	8	*	0,78	0,81	0,995125	0,995125	0,001413492	0,004462464	Jaspar_2020_MA0099.2-FOS::JUN			
chr2L	7E+06	7E+06	12	*	0,85	0,89	0,968864	0,970636	4,24951E-05	0,000465779	Jaspar_2020_MA0099.3-FOS::JUN	AP1 BS γ2		
chr2L	7E+06	7E+06	12	*	0,89	0,89	0,958	0,958	3,50105E-05	5,62669E-05	MEME_fly_factor_survey-Ap1_FBgn0001291			
chr2L	7E+06	7E+06	12	*	0,89	0,89	0,958	0,958	3,50105E-05	5,62669E-05	MEME_fly_factor_survey-Ap1_FBgn0001297			
chr2L	7E+06	7E+06	10	*	0,95	1	0,987278	0,988889	1,24882E-05	0,00021105	Jaspar_2020_MA0099.1-JUN::FOS			AP1-2*
chr2L	7E+06	7E+06	18	*	0,82	1	0,846075	0,994	4,43146E-05	0,011828058	Jaspar_2020_MA0099.2-FOS::JUN			
chr2L	7E+06	7E+06	9	*	0,72	0,72	0,718333	0,718333	0,006824582	0,006824582	Jaspar_2020_MA0099.1-JUN::FOS			
chr2L	7E+06	7E+06	9	*	0,73	0,73	0,999556	0,999556	0,005375121	0,005375121	Jaspar_2020_MA0099.1-JUN::FOS	AP1 BS γ3		
chr2L	7E+06	7E+06	25	*	0,75	0,86	0,999425	1	0,000842394	0,011828058	Jaspar_2020_MA0099.2-FOS::JUN			AP1-3*
chr2L	7E+06	7E+06	12	*	0,75	0,78	0,999833	0,999833	0,000763145	0,002841477	MEME_fly_factor_survey-Ap1_FBgn0001291	AP1 BS γ4		
chr2L	7E+06	7E+06	12	*	0,75	0,78	0,999833	0,999833	0,000763145	0,002841477	MEME_fly_factor_survey-Ap1_FBgn0001297			
chr2L	7E+06	7E+06	12	*	0,78	0,83	0,999909	1	0,000224974	0,002269529	Jaspar_2020_MA0099.3-FOS::JUN			
chr2L	7E+06	7E+06	10	*	0,82	0,84	1	1	0,000709953	0,001599528	Jaspar_2020_MA0099.1-JUN::FOS			
chr2L	7E+06	7E+06	8	*	0,70	0,7	1	1	0,011828058	0,011828058	Jaspar_2020_MA0099.2-FOS::JUN	AP1 BS γ5		
chr2L	7E+06	7E+06	9	*	0,75	0,75	1	1	0,004083219	0,004083219	Jaspar_2020_MA0099.1-JUN::FOS			
chr2L	7E+06	7E+06	11	*	0,74	0,74	1	1	0,001850203	0,001850203	Jaspar_2020_MA0099.3-FOS::JUN	AP1 BS γ6		
chr2L	7E+06	7E+06	12	*	0,74	0,74	1	1	0,001931829	0,001931829	MEME_fly_factor_survey-Ap1_FBgn0001291			
chr2L	7E+06	7E+06	12	*	0,74	0,74	1	1	0,001931829	0,001931829	MEME_fly_factor_survey-Ap1_FBgn0001297			
chr2L	7E+06	7E+06	9	*	0,77	0,77	1	1	0,002410011	0,002410011	Jaspar_2020_MA0099.1-JUN::FOS			
chr2L	7E+06	7E+06	8	*	0,71	0,71	1	1	0,009428202	0,009428202	Jaspar_2020_MA0099.2-FOS::JUN			
chr2L	7E+06	7E+06	8	*	0,70	0,7	1	1	0,011828058	0,011828058	Jaspar_2020_MA0099.2-FOS::JUN	AP1 BS γ7		
chr2L	7E+06	7E+06	9	*	0,72	0,72	1	1	0,006824582	0,006824582	Jaspar_2020_MA0099.1-JUN::FOS	AP1 BS γ8		
chr2L	7E+06	7E+06	8	*	0,76	0,76	1	1	0,003440731	0,003440731	Jaspar_2020_MA0099.2-FOS::JUN			
chr2L	7E+06	7E+06	11	*	0,71	0,71	0,998909	0,998909	0,00338315	0,00338315	Jaspar_2020_MA0099.3-FOS::JUN	AP1 BS γ9		
chr2L	7E+06	7E+06	10	*	0,74	0,76	0,998833	0,999	0,003164505	0,006824582	Jaspar_2020_MA0099.1-JUN::FOS			
chr2L	7E+06	7E+06	8	*	0,79	0,84	0,998875	0,998875	0,001068753	0,005905707	Jaspar_2020_MA0099.2-FOS::JUN			
chr2L	7E+06	7E+06	8	*	0,70	0,7	0,999625	0,999625	0,011828058	0,011828058	Jaspar_2020_MA0099.2-FOS::JUN	AP1 BS γ10		
chr2L	7E+06	7E+06	9	*	0,73	0,73	0,814111	0,814111	0,005375121	0,005375121	Jaspar_2020_MA0099.1-JUN::FOS	AP1 BS γ11		
chr2L	7E+06	7E+06	8	*	0,73	0,73	0,790875	0,790875	0,007121135	0,007121135	Jaspar_2020_MA0099.2-FOS::JUN			
chr2L	7E+06	7E+06	12	*	0,79	0,79	1	1	0,000607474	0,000763145	MEME_fly_factor_survey-Ap1_FBgn0001291	AP1 BS γ12		
chr2L	7E+06	7E+06	12	*	0,79	0,79	1	1	0,000607474	0,000763145	MEME_fly_factor_survey-Ap1_FBgn0001297			
chr2L	7E+06	7E+06	12	*	0,78	0,82	1	1	0,000281634	0,001850203	Jaspar_2020_MA0099.3-FOS::JUN			
chr2L	7E+06	7E+06	10	*	0,81	0,86	1	1	0,000389839	0,003164505	Jaspar_2020_MA0099.1-JUN::FOS			
chr2L	7E+06	7E+06	8	*	0,82	0,86	1	1	0,000842394	0,002640987	Jaspar_2020_MA0099.2-FOS::JUN			AP1-4*
chr2L	7E+06	7E+06	18	*	0,77	0,81	0,999481	1	0,001068988	0,007809067	Jaspar_2020_MA0099.1-JUN::FOS	AP1 BS γ13		
chr2L	7E+06	7E+06	8	*	0,78	0,83	0,999125	0,999125	0,001161335	0,007121135	Jaspar_2020_MA0099.2-FOS::JUN			
chr2L	7E+06	7E+06	12	*	0,73	0,73	1	1	0,002300481	0,002300481	MEME_fly_factor_survey-Ap1_FBgn0001291			
chr2L	7E+06	7E+06	12	*	0,73	0,73	1	1	0,002300481	0,002300481	MEME_fly_factor_survey-Ap1_FBgn0001297			
chr2L	7E+06	7E+06	12	*	0,74	0,75	1	1	0,004462464	0,007121135	Jaspar_2020_MA0099.2-FOS::JUN			
chr2L	7E+06	7E+06	11	*	0,70	0,7	0,910455	0,910455	0,00408307	0,00408307	Jaspar_2020_MA0099.3-FOS::JUN	AP1 BS γ14		
chr2L	7E+06	7E+06	12	*	0,70	0,7	0,917917	0,917917	0,004240646	0,004240646	MEME_fly_factor_survey-Ap1_FBgn0001291			
chr2L	7E+06	7E+06	12	*	0,70	0,7	0,917917	0,917917	0,004240646	0,004240646	MEME_fly_factor_survey-Ap1_FBgn0001297			
chr2L	7E+06	7E+06	8	*	0,73	0,73	0,997	0,997	0,007121135	0,007121135	Jaspar_2020_MA0099.2-FOS::JUN			
chr2L	7E+06	7E+06	12	*	0,71	0,71	1	1	0,003389975	0,003389975	MEME_fly_factor_survey-Ap1_FBgn0001291	AP1 BS γ15		
chr2L	7E+06	7E+06	12	*	0,71	0,71	1	1	0,003389975	0,003389975	MEME_fly_factor_survey-Ap1_FBgn0001297			
chr2L	7E+06	7E+06	8	*	0,75	0,78	1	1	0,002640987	0,008114574	Jaspar_2020_MA0099.2-FOS::JUN			
chr2L	7E+06	7E+06	9	*	0,80	0,8	1	1	0,001599528	0,001599528	Jaspar_2020_MA0099.1-JUN::FOS			
chr2L	7E+06	7E+06	12	*	0,80	0,81	1	1	0,000363291	0,000608471	Jaspar_2020_MA0099.3-FOS::JUN	AP1 BS γ16		
chr2L	7E+06	7E+06	12	*	0,81	0,85	1	1	0,000128372	0,001199109	MEME_fly_factor_survey-Ap1_FBgn0001291			
chr2L	7E+06	7E+06	12	*	0,81	0,85	1	1	0,000128372	0,001199109	MEME_fly_factor_survey-Ap1_FBgn0001297			
chr2L	7E+06	7E+06	10	*	0,89	0,93	1	1	7,40965E-05	0,000581117	Jaspar_2020_MA0099.1-JUN::FOS			
chr2L	7E+06	7E+06	8	*	0,91	0,93	1	1	0,000111099	0,000401536	Jaspar_2020_MA0099.2-FOS::JUN			AP1-5*
chr2L	7E+06	7E+06	8	*	0,70	0,7	1	1	0,011828058	0,011828058	Jaspar_2020_MA0099.2-FOS::JUN	AP1 BS γ17		
chr2L	7E+06	7E+06	9	*	0,72	0,72	1	1	0,006824582	0,006824582	Jaspar_2020_MA0099.1-JUN::FOS	AP1 BS γ18		
chr2L	7E+06	7E+06	8	*	0,70	0,7	1	1	0,011828058	0,011828058	Jaspar_2020_MA0099.2-FOS::JUN			
chr2L	7E+06	7E+06	9	*	0,70	0,7	1	1	0,009040818	0,009040818	Jaspar_2020_MA0099.1-JUN::FOS	AP1 BS γ19		
chr2L	7E+06	7E+06	8	*	0,75	0,75	1	1	0,004462464	0,004462464	Jaspar_2020_MA0099.2-FOS::JUN			

*Depicted in Fig 5 &

Region	Start	End	Num bp	AP1 Score
				1-0,91
α	7323135	7324749	1614	0,90-0,85
β	7324770	7325443	673	0,84-0,80
γ	7325445	7326565	1120	0,79-0,75
δ	7326565	7327855	1290	0,74-0,70

Supplementary Table 5. AP1-predicted binding sites.

Table containing information on the bioinformatically-predicted AP1 binding sites identified in this enhancer (genomic coordinates and statistical parameters on conservation and match).

Figure	Genotype	n Wing to notum transformation	n total heminotas	% wing to notum transformation
1b	*Δwg*^{1}*/ΔBRV118*	83	102	81,4%
	Δwg^{1}*/BSC226*	217	250	86,8%
	Δwg^{1}*/BSC291*	41	44	93,2%
	Δwg^{1}*/BSC324*	137	142	96,5%
	ΔBRV118/ΔBRV118	324	470	68,9%
	ΔBRV118/BSC226	73	82	89,0%
	ΔBRV118/BSC291	21	22	95,5%
	ΔBRV118/BSC324	317	318	99,7%
	CX4/Δwg^{1}	33	140	23,6%
	CX4/ΔBRV118	26	78	33,3%
	CX3/Δwg^{1}	126	242	52,1%
	CX3/ΔBRV118	213	270	78,9%
	wnt6 KO/wnt6 KO	0	742	0%
	wnt6 KO/Δwg^{1}	0	360	0%
	wnt6 KO/ΔBRV118	0	469	0%
	wnt6 KO/BSC226	0	118	0%
	wnt6 KO/BSC291	0	200	0%
	wnt6 KO/BSC324	0	142	0%
2j	*Sd>EGFP-i*	0	526	0%
	Sd>ptc	11	244	4,50%
4c	*Δβγ/Δβγ*	437	550	79%
	Δβγ/ΔBRV118	629	734	86%
	Δγ/Δγ	5	1280	0,39%
	Δγ/Δβγ	59	448	12%
	Δγ/ΔBRV118	58	156	37%
	Δγ-590/Δγ-590	0	1196	0%
	Δγ-590/Δβγ	1	512	0,20%
	Δγ-590/ΔBRV118	4	242	1,65%
	Δβ/Δβ	0	382	0%
	Δβ/Δγ	0	432	0%
	Δβ/Δβγ	0	324	0%
	Δβ/ΔBRV118	0	378	0%
	w^{1118}*/w*^{1118}	0	944	0%

Supplementary Table 6. Wing to notum transformation phenotype: quantification.

Table containing the percentages of wing to notum transformation observed in the indicated genotypes. Number of heminota quantified and the figure legend where these data appear are indicated.

Fig	Parameter	Genotype	n total	mean±SD	Effect size (lm coefficient)	95% Conf. interval	Degrees of freedom	p value	Significance	Statistical test
6d	Wg signal Intensity (a.u.)	*+/+;rn>egr,tub-gal80* ^{ts}	29	9,124 ±2,518						log2 transformation + linear regression adjusting by Experimental batch + Dunnett's multiple comparison (two-tailed test)
		Δβ/Δβ;rn>egr,tub-gal80 ^{ts}	25	6,094 ±1,318	-0,5623	[-0.8476,-0.2769]	101	0,00002179	***	
		Δγ/Δγ;rn>egr,tub-gal80 ^{ts}	25	6,728 ±1,728	-0,426	[-0.7117,-0.1403]	101	0,001596	**	
		Δβγ/Δβγ;rn>egr,tub-gal80 ^{ts}	28	5,217 ±3,066	-0,8956	[-1.1734,-0.6178]	101	4,5793E-11	***	
6d	number of pH3 cells / area (a.u.)	*+/+;rn>egr,tub-gal80ts*	29	51,18 ±17,10						log2 transformation + linear regression adjusting by Experimental batch + Dunnett's multiple comparison (two-tailed test)
		Δβ/Δβ;rn>egr,tub-gal80 ^{ts}	25	42,29 ±12,74	-0,2363	[-0.5649, 0.0922]	100	0,21476	NS	
		Δγ/Δγ;rn>egr,tub-gal80 ^{ts}	24	34 ±15,86	-0,62	[-0.9493, -0.2845]	100	0,00007299	***	
		Δβγ/Δβγ;rn>egr,tub-gal80 ^{ts}	28	31,23 ±10,37	-0,7045	[-1.0244, -0.3847]	100	0,000002022	***	
6d	EdU / area (a.u.)	*+/+;rn>egr,tub-gal80ts*	24	0,14 ±0,07						Boxcox transformation + linear regression adjusting by Experimental batch + Dunnett's multiple comparison (two-tailed test)
		Δβ/Δβ;rn>egr,tub-gal80 ^{ts}	27	0,11 ±0,08	-0,5311	[-1.3970, 0.3347]	94	0,2340659	NS	
		Δγ/Δγ;rn>egr,tub-gal80 ^{ts}	23	0,12 ±0,07	-0,57	[-1.4665, 0.3270]	94	0,5386233	NS	
		Δβγ/Δβγ;rn>egr,tub-gal80 ^{ts}	26	0,07 ±0,06	-1,2495	[-2.1185, -0.3806]	94	0,0010462	**	
6i	number of pH3 cells / area (a.u.)	*rn>egr,EGFP-i,tub-gal80* ^{ts}	18	46,74 ±17,26						Linear regression adjusting by Experimental batch (two-tailed test)
		rn>egr,wnt6-i,tub-gal80 ^{ts}	25	31,78 ±10,09	-15,1691	[-23.4537, -6.8845]	39	0,00065694	***	
7e	Wing disc area	*+/+;hh>rod-i,p35,tub-gal80* ^{ts}	13	172400 ±38440						Linear regression adjusting by Experimental batch + Dunnett's multiple comparison (two-tailed test)
		Δβ/Δβ;hh>rod-i,p35,tub-gal80 ^{ts}	32	119900 ±30110	-52476,71	75926.09, -29027.32	82	4,2649E-06	***	
		Δγ/Δγ;hh>rod-i,p35,tub-gal80 ^{ts}	29	142000 ±27940	-30388,65	54185.85, -6591.44	82	0,0091812	**	
		Δβγ/Δβγ;hh>rod-i,p35,tub-gal80 ^{ts}	12	113600 ±24960	-58834,12	87375.80, -30292.43	82	0,000016546	***	
7e	Wg signal Intensity	*+/+;hh>rod-i,p35,tub-gal80* ^{ts}	13	15,72 ±4,166						log2 transformation + linear regression adjusting by Experimental batch + Dunnett's multiple comparison (two-tailed test)
		Δβ/Δβ;hh>rod-i,p35,tub-gal80 ^{ts}	32	8,489 ±3,569	-0,9777	[-1.3690, -0.5865]	80	1,1351E-07	***	
		Δγ/Δγ;hh>rod-i,p35,tub-gal80 ^{ts}	29	8,557 ±2,802	-0,9132	[-1.3105, -0.5160]	80	7,4461E-06	***	
		Δβγ/Δβγ;hh>rod-i,p35,tub-gal80 ^{ts}	12	5,480 ±2,151	-1,6788	[-2.1646, -1.1930]	80	2,5145E-12	***	
7g	Wing disc area	*+/+;hh>rod-i,p35,tub-gal80* ^{ts}	28	173100 ±46270						Linear regression adjusting by Experimental batch (two-tailed test)
		wnt 6KO/wnt6 KO;hh>rod-i,p35,tub-gal80 ^{t}	33	131200 ±20090	-41539,13	-59314.9, -23763.35	57	0,000018162	***	

Fig.	Genotype	Regenerated wings	Non-Regenerated	n total	Percentage regenerated wings	Effect size (lm coefficient)	95% Conf. interval	Degrees of freedom	p value	Significance	Statistical test
6g	*+/+;rn>egr,tub-gal80* ^{ts}	288	51	339	85%						Logistic regression adjusting by Experimental batch + Dunnett's multiple comparison (two-tailed
	Δβ/Δβ;rn>egr,tub-gal80 ^{ts}	82	89	171	48%	-2,0658	[-2.6013, -1.5303]	27	2,22E-16	***	
	Δβ/Δγ;rn>egr,tub-gal80 ^{ts}	48	89	137	35%	-2,814	[-3.4107, -2.217]	27	2,22E-16	***	
6g	*+/+;rn>egr,tub-gal80* ^{ts}	150	39	189	79%						Logistic regression adjusting by Experimental batch (two-tailed test)
	Δγ/Δ;rn>egr,tub-gal80 ^{ts}	91	54	145	63%	-3,3638	[-2.3737, -0.9901]	7	1,89E-06	***	
6g	*+/+;rn>egr,tub-gal80* ^{ts}	336	48	384	88%						Logistic regression adjusting by Experimental batch (two-tailed test)
	Δβγ/Δβγ;rn>egr,tub-gal80 ^{ts}	27	50	77	35%	-2,6134	[-3.1929, -2.034]	17	2,22E-16	***	
6h	*rn>egr,EGFP,tub-gal80* ^{ts}	232	46	278	83%						Logistic regression adjusting by Experimental batch (two-tailed test)
	rn>egr,wnt6-i,tub-gal80 ^{ts}	58	78	136	43%	-2,0235	[-2.5183, -1.5287]	14	1,11E-15	***	

Supplementary Table 7. Summary of n and p-values.

Table containing the parameters that have been quantified, the figures where these quantifications are shown, and the statistical details.

Supplementary Table 8. Resources table.

REAGENT or RESOURCE	SOURCE	IDENTIFIER
**Antibodies**		
mouse anti-dMMP1 (14A3D2)	Developmental Studies Hybridoma bank	RRID: AB_579782
goat polyclonal anti-GFP (ab6673)	Abcam	Code: ab6673
rabbit anti-β-galactosidase (0855976)	Cappel (MP Biochemicals)	Code: 0855976
mouse anti-β-galactosidase (40.1a)	Developmental Studies Hybridoma bank	RRID: AB_2314509
rabbit anti-phospho-Histone H3 (pH3)	Cell Signaling	RRID: AB_331535
rat anti-Ci (2A1)	Developmental Studies Hybridoma bank	RRID: AB_2109711
mouse anti-Wg (4D4)	Developmental Studies Hybridoma bank	RRID: AB_528512
mouse anti-Nubbin (nub2D4)	Developmental Studies Hybridoma bank	RRID: AB_2722119
rabbit anti-Tsh	[1]	N/A
Click-iT™ Plus EdU Alexa Fluor™ 647 Imaging Kit	Invitrogen	Code: C10640
Cy2 AffiniPure Donkey Anti-Rat IgG (H+L)	Jackson ImmunoResearch	Code: 712-225-150
Cy2 AffiniPure Donkey Anti-Goat IgG (H+L)	Jackson ImmunoResearch	Code: 705-225-147
Cy5 AffiniPure Donkey Anti-Mouse IgG (H+L)	Jackson ImmunoResearch	Code: 715-175-151
Cy5 AffiniPure Donkey Anti-Rabbit IgG (H+L)	Jackson ImmunoResearch	Code: 711-175-152
Cy3 AffiniPure Donkey Anti-Rat IgG (H+L)	Jackson ImmunoResearch	Code: 712-165-153
Cy3 AffiniPure Donkey Anti-Mouse IgG (H+L)	Jackson ImmunoResearch	Code: 715-165-150
Cy3 AffiniPure Donkey Anti-Rabbit IgG (H+L)	Jackson ImmunoResearch	Code: 711-165-152
**Chemicals, Peptides and Recombinant Proteins**		
DAPI	Sigma Aldrich	Code: 28718-90-3
**Oligonucleotides**		
CTTCGGATAGGAAGGTATTGCGAC	Invitrogen	*Beta* CRISPR_Down-Fwd

AAACGTCGCAATACCTTCCTATCC	Invitrogen	*Beta* CRISPR_Down-Rev
CTTCGGCATATTGGACTGTGTTCG	Invitrogen	*Beta_Gamma* CRISPR_Up-Fwd
AAACCGAACACAGTCCAATATGCC	Invitrogen	*Beta_Gamma* CRISPR_Up-Rev
CTTCGTTCCCAATCTCAAAAGATGT	Invitrogen	*Gamma* CRISPR_Up-Fwd
AAACACATCTTTTGAGATTGGGAAC	Invitrogen	*Gamma* CRISPR_Up-Rev
CTTCGTCGAGTGCACCGATCTTCCC	Invitrogen	*Gamma* CRISPR_Down-Fwd
AAACGGGAAGATCGGTGCACTCGAC	Invitrogen	*Gamma* CRISPR_Down-Rev
CTTCTCTGGCGATCCGGGGAGCTA	Invitrogen	*Gamma*590 CRISPR_Up-Fwd
AAACTAGCTCCCCGGATCGCCAGA	Invitrogen	*Gamma*590 CRISPR_Up-Rev
CTTCTCGAGTGCACCGATCTTCCC	Invitrogen	*Gamma*590 CRISPR_Down-Fwd
AAACGGGAAGATCGGTGCACTCGA	Invitrogen	*Gamma*590 CRISPR_Down-Rev
GTC GAA ATT AAG AGA CCA CAC GCA	Invitrogen	*Gamma* Ci1mut CRISPR_Fwd
AAA CTG CGT GTG GTC TCT TAA TTT	Invitrogen	*Gamma* Ci1mut CRISPR_Rev
GCGGAATTCTAATGTTATAGTATTTCTGCT	Invitrogen	wg-*Alpha*-Fwd (EcoRI)
CCGGGTACCCTACTTTATAAATTTACATTA	Invitrogen	wg-*Alpha*-Rev (KpnI)
GCGGAATTCTTAAACCGATTTTATTACCCA	Invitrogen	wg-*Beta*-Fwd (EcoRI)
CCGGGTACCAAGGTTTTATATCTAACCTAC	Invitrogen	wg-*Beta*-Rev (KpnI)
CGGGGTACCGTTTTATTATATTTCGACAAA	Invitrogen	wg-*Gamma*-Fwd (KpnI)
ATAGTTTAGCGGCCGCTATGAGTACTTAA CTAAATGT	Invitrogen	wg-*Gamma*-Rev (NotI)
GCGGAATTCGTAAAACAGTTTTATTTTGGG	Invitrogen	wg-*Delta*-Fwd (EcoRI)
CCGGGTACCATTTATAAACGACGTATAGT T	Invitrogen	wg-*Delta*-Rev (KpnI)
ATATGGTACCTTTTGTTTTATTATATTTCGA CAAAATCG	Invitrogen	wg-*GammaDelta*-Fw (KpnI)
ATAAGAATGCGGCCGCACATTTCATATGT CAACCCTTCGTTT	Invitrogen	wg-*GammaDelta*-Rev (NotI)
CGG ATC CGG GGA GCT ACG GAG TTG CGG AGC AGC GTT	Invitrogen	wg-*Gamma*590-Fwd
GGGGTACCAGGCATTGCGACAGGAGCTA TGGGAGGTTTTT	Invitrogen	wg- *Gamma*630-Fwd (KpnI)

ATAAGAATGCGGCCGCTCCCCGGATCGC CAGATGCCCAGA	Invitrogen	wg- *Gamma*630-Rev (NotI)
CGGGGTACCAATTCTCGTGAACCTCCCAG CACATCT	Invitrogen	spdFlag CiS2-Fwd
ATAGTTTAGCGGCCGCACTACAATTCTAG TTAGTTT	Invitrogen	spdFlag CiS2-Fwd
CGGGTACCTGACGAATAGCCCATAGCTCT G	Invitrogen	spdFlag350 CiS2-Fwd
ATAGTTTAGCGCGGCCGCATATTAGCATG ATAAGT	Invitrogen	spdFlag350 CiS2-Rv
GGTACCTTCAAGTTTCTTTCCCCAACCTTA AGT	Invitrogen	wnt6-1st Intron CiBS1-Fwd
ATAGTTTAGCGGCCGCCTTTAGTTCGCTTT TAATGC	Invitrogen	wnt6-1st Intron CiBS1-Rev
CGGGGTACCGTTTTATTATATTTCGACAAA	Invitrogen	**PCR1:** wg-*Gamma*-Fwd
AAATTAAGAGACTATACGCAAGGTGTGCT C	Invitrogen	**PCR1:** wg-*Gamma*Ci mut- Rev
GAGCACACCTTGCGTATAGTCTCTTAATTT	Invitrogen	**PCR2:** wg-*Gamma*Mut-Fwd
ATAGTTTAGCGGCCGCTATGAGTACTTAA CTAAATGT	Invitrogen	**PCR2:** wg-*Gamma*-Rev
CGGATCCGGGGAGCTACGGAGTTG CGGAGCAGCGTT	Invitrogen	wg-*Gamma*590-Fwd
ATAGTTTAGCGGCCGCTATGAGTACTTAA CTAAATGT	Invitrogen	wg-*Gamma*-Rev
**Experimental Models. Organisms/Strains**		
wg^1	Bloomington Drosophila Stock Center	RRID: BDSC_2978
ΔBRV118	[2]	RRID: BDSC_23676
Df(2L)BSC226	Bloomington Drosophila Stock Center	RRID: BDSC_9703
Df(2L)BSC291	Bloomington Drosophila Stock Center	RRID: BDSC_23676
Df(2L)BSC324	Bloomington Drosophila Stock Center	RRID: BDSC_24349
wg^{CX4}	Bloomington Drosophila Stock Center	RRID: BDSC_2980
wg^{CX3}	Bloomington Drosophila Stock Center	RRID: BDSC_2977

GFP-Wg	[3]	N/A
nlsGFP-DWnt6	[3]	N/A
wnt6^{KO}	[4]	RRID:BDSC_76311
wg^{02657} (wg-lacZ in the text)	Bloomington Drosophila Stock Center	RRID:BDSC_11205
wg^{1}-lacZ	generated in this work	N/A
BRV118-lacZ	[5]	N/A
sd-GAL4	Bloomington Drosophila Stock Center	RRID:BDSC_8609
UAS-EGFP	Bloomington Drosophila Stock Center	RRID:BDSC_9331
UAS-Hh-GFP	[6]	N/A
UAS-ptc	[6]	N/A
UAS-egfp^{RNAi}	Bloomington Drosophila Stock Center	RRID:BDSC_35786
UAS-vn-argos	[7]	N/A
α-lacZ	generated in this work	N/A
β-lacZ	generated in this work	N/A
γ-lacZ	generated in this work	N/A
δ-lacZ	generated in this work	N/A
γδ -lacZ	generated in this work	N/A
γ-630-lacZ	generated in this work	N/A
γ-590-lacZ	generated in this work	N/A
γ-590(Ci)-lacZ*	generated in this work	N/A
γ (Ci)-lacZ*	generated in this work	N/A
Δγ-Ci-BS	generated in this work	N/A
Δγ-590	generated in this work	N/A
Δγ	generated in this work	N/A
Δβ	generated in this work	N/A
Δβγ	generated in this work	N/A
rn-Gal4,tub-Gal80^{ts},UAS-egr	[8]	N/A

*sal-lexA, tubg80^ts^*	[9]	N/A
lexO-rpr	[9]	N/A
*UAS-wnt6^RNAi^*	VDRC Stock Center	RRID:VDRC_26669
*hh-Gal4,tub-Gal80^ts^*	[10]	N/A
*UAS-rod^RNAi^*	VDRC Stock Center	RRID:VDRC_16152
UAS-p35	Bloomington Drosophila Stock Center	RRID:BDSC_5073
UAS-bskDN	Bloomington Drosophila Stock Center	RRID:BDSC_6409
en-Gal4	Bloomington Drosophila Stock Center	RRID:BDSC_1973
*dpp^disk^-gal4 (dpp-Gal4 in the text) tub-Gal80^ts^*	[11]	N/A
UAS-wg-GFP	[12]	N/A
UAS-wnt6	FlyORF	RRID: F003540
spdFlag-lacZ	generated in this work	
wnt6-intron-lacZ	generated in this work	
y^1v^1P{nos-phiC31\int.NLS}X; P{CaryP}attP40	Bloomington Drosophila Stock Center	RRID:BDSC_25709
v^1; Sco / SM6a	Bloomington Drosophila Stock Center	RRID:BDSC_137
w^{1118}	Bloomington Drosophila Stock Center	RRID:BDSC_3605
**Software and Algorithms**		
Fiji	Fiji	https://fiji.sc/
Excel	Microsoft Excel 2016	N/A
GraphPad Prism 7 Project	GraphPad	RRID:SCR_002798

Supplementary References

1. Wu, J., and Cohen, S.M. (2002). Repression of Teashirt marks the initiation of wing development. Development *129*, 2411–8.

2. Harris, R.E., Setiawan, L., Saul, J., and Hariharan, I.K. (2016). Localized epigenetic silencing of a damage-activated WNT enhancer limits regeneration in mature Drosophila imaginal discs. Elife *5*.

3. Yu, J.J.S., Maugarny-Calès, A., Pelletier, S., Alexandre, C., Bellaiche, Y., Vincent, J.P., and McGough,

I.J. (2020). Frizzled-Dependent Planar Cell Polarity without Secreted Wnt Ligands. Dev. Cell *54*, 583-592.e5.

4. Doumpas, N., Jékely, G., and Teleman, A.A. (2013). Wnt6 is required for maxillary palp formation in Drosophila. BMC Biol. *11*, 104.

5. Schubiger, M., Sustar, A., and Schubiger, G. (2010). Regeneration and transdetermination: the role of wingless and its regulation. Dev Biol *347*, 315–324.

6. Callejo, A., Culi, J., and Guerrero, I. (2008). Patched, the receptor of Hedgehog, is a lipoprotein receptor. Proc. Natl. Acad. Sci. U. S. A. *105*, 912–917.

7. Wang, S.H., Simcox, A., and Campbell, G. (2000). Dual role for Drosophila epidermal growth factor receptor signaling in early wing disc development. Genes Dev *14*, 2271–6.

8. Smith-Bolton, R.K., Worley, M.I., Kanda, H., and Hariharan, I.K. (2009). Regenerative growth in Drosophila imaginal discs is regulated by Wingless and Myc. Dev Cell *16*, 797–809.

9. Santabárbara-Ruiz, P., López-Santillán, M., Martínez-Rodríguez, I., Binagui-Casas, A., Pérez, L., Milán, M., Corominas, M., and Serras, F. (2015). ROS-Induced JNK and p38 Signaling Is Required for Unpaired Cytokine Activation during Drosophila Regeneration. PLOS Genet. *11*, e1005595.

10. Muzzopappa, M., Murcia, L., and Milán, M. (2017). Feedback amplification loop drives malignant growth in epithelial tissues. Proc. Natl. Acad. Sci. *114*, E7291–E7300.

11. Staehling-Hampton, K., Hoffmann, F.M., Baylies, M.K., Rushton, E., and Bate, M. (1994). dpp induces mesodermal gene expression in Drosophila. Nature *372*, 783–786.

12. Pfeiffer, S., Ricardo, S., Manneville, J.B., Alexandre, C., and Vincent, J.P. (2002). Producing cells retain and recycle Wingless in Drosophila embryos. Curr Biol *12*, 957–62.
