# Supplementary material for: A single WNT enhancer drives specification and regeneration of the Drosophila wing
Source: Nat Commun. 2022 Aug 22;13:4794. doi: 10.1038/s41467-022-32400-2 (PMC9395397; doi:10.1038/s41467-022-32400-2)
Supplement: Supplementary file 3 — Reporting Summary [file 41467_2022_32400_MOESM3_ESM.pdf]

Corresponding author(s): NCOMMS-22-04013B

Last updated by author(s): Jul 21, 2022

Reporting Summary

Nature Portfolio wishes to improve the reproducibility of the work that we publish. This form provides structure for consistency and transparency in reporting. For further information on Nature Portfolio policies, see our Editorial Policies and the Editorial Policy Checklist.

Statistics

For all statistical analyses, confirm that the following items are present in the figure legend, table legend, main text, or Methods section.

n/a	Confirmed	
☐	☒	The exact sample size (*n*) for each experimental group/condition, given as a discrete number and unit of measurement
☐	☒	A statement on whether measurements were taken from distinct samples or whether the same sample was measured repeatedly
☐	☒	The statistical test(s) used AND whether they are one- or two-sided<br>*Only common tests should be described solely by name; describe more complex techniques in the Methods section.*
☒	☐	A description of all covariates tested
☐	☒	A description of any assumptions or corrections, such as tests of normality and adjustment for multiple comparisons
☐	☒	A full description of the statistical parameters including central tendency (e.g. means) or other basic estimates (e.g. regression coefficient) AND variation (e.g. standard deviation) or associated estimates of uncertainty (e.g. confidence intervals)
☐	☒	For null hypothesis testing, the test statistic (e.g. *F*, *t*, *r*) with confidence intervals, effect sizes, degrees of freedom and *P* value noted<br>*Give P values as exact values whenever suitable.*
☒	☐	For Bayesian analysis, information on the choice of priors and Markov chain Monte Carlo settings
☒	☐	For hierarchical and complex designs, identification of the appropriate level for tests and full reporting of outcomes
☒	☐	Estimates of effect sizes (e.g. Cohen's *d*, Pearson's *r*), indicating how they were calculated

Our web collection on statistics for biologists contains articles on many of the points above.

Software and code

Policy information about availability of computer code

Data collection	*Provide a description of all commercial, open source and custom code used to collect the data in this study, specifying the version used OR state that no software was used.*
Data analysis	Fiji Fiji https://fiji.sc/<br>Excel Microsoft Excel 2016 N/A<br>GraphPad Prism 7 Project GraphPad RRID:SCR_002798

For manuscripts utilizing custom algorithms or software that are central to the research but not yet described in published literature, software must be made available to editors and reviewers. We strongly encourage code deposition in a community repository (e.g. GitHub). See the Nature Portfolio guidelines for submitting code & software for further information.

Data

Policy information about availability of data

All manuscripts must include a data availability statement. This statement should provide the following information, where applicable:

- Accession codes, unique identifiers, or web links for publicly available datasets
- A description of any restrictions on data availability
- For clinical datasets or third party data, please ensure that the statement adheres to our policy

Source data are provided with this paper.

Human research participants

Policy information about studies involving human research participants and Sex and Gender in Research.

Reporting on sex and gender	*Use the terms sex (biological attribute) and gender (shaped by social and cultural circumstances) carefully in order to avoid confusing both terms. Indicate if findings apply to only one sex or gender; describe whether sex and gender were considered in study design whether sex and/or gender was determined based on self-reporting or assigned and methods used. Provide in the source data disaggregated sex and gender data where this information has been collected, and consent has been obtained for sharing of individual-level data; provide overall numbers in this Reporting Summary. Please state if this information has not been collected. Report sex- and gender-based analyses where performed, justify reasons for lack of sex- and gender-based analysis.*
Population characteristics	*Describe the covariate-relevant population characteristics of the human research participants (e.g. age, genotypic information, past and current diagnosis and treatment categories). If you filled out the behavioural & social sciences study design questions and have nothing to add here, write "See above."*
Recruitment	*Describe how participants were recruited. Outline any potential self-selection bias or other biases that may be present and how these are likely to impact results.*
Ethics oversight	*Identify the organization(s) that approved the study protocol.*

Note that full information on the approval of the study protocol must also be provided in the manuscript.

Field-specific reporting

Please select the one below that is the best fit for your research. If you are not sure, read the appropriate sections before making your selection.

☒ Life sciences ☐ Behavioural & social sciences ☐ Ecological, evolutionary & environmental sciences

For a reference copy of the document with all sections, see nature.com/documents/nr-reporting-summary-flat.pdf

Life sciences study design

All studies must disclose on these points even when the disclosure is negative.

Sample size	At least 10 samples per genotype and experiment. Sample size was quantified manually.
Data exclusions	None
Replication	Three times at least. All attempts at replication were successful.
Randomization	For those quantifications performed in larval wing primordia, larvae of the corresponding genotypes were selected at random from the vial and wing discs were dissected to perfom the immunostainings and the corresponding quantifications. For those quantifications performed in the resulting adults subjected to experimental or genetic manipulations, all adults that hatched were scored, so no need for randomization in this case.
Blinding	Quantification was carried following objective criteria such as the use of Macros in Fiji or clear-cut quantitative data (number of mitotic figures, number of flies with wings, number of wings not regenerated). As such, blinding, which is not commonly used in our field, was applied only for the statistic analysis which was performed by a statistician of the IRB Biostatistics Facility.

Reporting for specific materials, systems and methods

We require information from authors about some types of materials, experimental systems and methods used in many studies. Here, indicate whether each material, system or method listed is relevant to your study. If you are not sure if a list item applies to your research, read the appropriate section before selecting a response.

Materials & experimental systems

n/a	Involved in the study
☐	☒ Antibodies
☒	☐ Eukaryotic cell lines
☒	☐ Palaeontology and archaeology
☐	☒ Animals and other organisms
☒	☐ Clinical data
☒	☐ Dual use research of concern

Methods

n/a	Involved in the study
☒	☐ ChIP-seq
☒	☐ Flow cytometry
☒	☐ MRI-based neuroimaging

Antibodies

Antibodies used

mouse anti-dMMP1 (14A3D2) Developmental Studies Hybridoma bank RRID: AB_579782
goat polyclonal anti-GFP (ab6673) Abcam Code: ab6673
rabbit anti-β-galactosidase (0855976) Cappel (MP Biochemicals) Code: 0855976
mouse anti-β-galactosidase (40.1a) Developmental Studies Hybridoma bank RRID: AB_2314509

rabbit anti-phospho-Histone H3 (pH3) Cell Signaling RRID: AB_331535
rat anti-Ci (2A1) Developmental Studies Hybridoma bank RRID: AB_2109711
mouse anti-Wg (4D4) Developmental Studies Hybridoma bank RRID: AB_528512

mouse anti-Nubbin (nub2D4) Developmental Studies Hybridoma bank RRID: AB_2722119
rabbit anti-Tsh 1
N/A
Click-iT™ Plus EdU Alexa Fluor™ 647 Imaging Kit Invitrogen Code: C10640
Cy2 AffiniPure Donkey Anti-Rat IgG (H+L) Jackson ImmunoResearch Code: 712-225-150
Cy2 AffiniPure Donkey Anti-Goat IgG (H+L) Jackson ImmunoResearch Code: 705-225-147
Cy5 AffiniPure Donkey Anti-Mouse IgG (H+L) Jackson ImmunoResearch Code: 715-175-151
Cy5 AffiniPure Donkey Anti-Rabbit IgG (H+L) Jackson ImmunoResearch Code: 711-175-152
Cy3 AffiniPure Donkey Anti-Rat IgG (H+L) Jackson ImmunoResearch Code: 712-165-153
Cy3 AffiniPure Donkey Anti-Mouse IgG (H+L) Jackson ImmunoResearch Code: 715-165-150
Cy3 AffiniPure Donkey Anti-Rabbit IgG (H+L) Jackson ImmunoResearch Code: 711-165-152

Validation

All antibodies used in this work have been validated by the publications described in Developmental Studies Hybridoma bank, Abcam, Cappel and Cell Signaling, and are of regular use in the Drosophila community.

Animals and other research organisms

Policy information about studies involving animals; ARRIVE guidelines recommended for reporting animal research, and Sex and Gender in Research

Laboratory animals

The following Drosophila strains at the larval (48-120 hours after egg laying) and adult stage (2-20 days after hatching) were used:
wg1
ΔBRV118
Df(2L)BSC226
Df(2L)BSC291
Df(2L)BSC324
wgCX4
wgCX3
GFP-Wg
nlsGFP-DWnt6
wnt6KO
wg02657 (wg-lacZ in the text)
wg1-lacZ
BRV118-lacZ
sd-GAL4
UAS-EGFP
UAS-Hh-GFP
UAS-ptc
UAS-egfpRNAi
UAS-vn-argos
α-lacZ
β-lacZ
γ-lacZ
δ-lacZ
γδ -lacZ

March 2021

γ-630-lacZ
γ-590-lacZ
γ-590(Ci*)-lacZ
γ (Ci*)-lacZ
Δγ-Ci-BS
Δγ-590
Δγ
Δβ
Δβγ
rn-Gal4,tub-Gal80ts,UAS-egr
sal-lexA, tubg80ts
lexO-rpr
UAS-wnt6RNAi
hh-Gal4,tub-Gal80ts
UAS-rodRNAi
UAS-p35
UAS-bskDN
en-Gal4
dppdisk-gal4 (dpp-Gal4 in the text) tub-Gal80ts
UAS-wg-GFP
UAS-wnt6
spdFlag-lacZ
wnt6-intron-lacZ
y1v1P{nos-phiC31\int.NLS}X; P{CaryP}attP40
v1; Sco / SM6a
w1118

Wild animals	No wild type animals were used in this study.
Reporting on sex	NA
Field-collected samples	NA
Ethics oversight	This study did not require an ethical approval.

Note that full information on the approval of the study protocol must also be provided in the manuscript.
